# The usefulness of preoperative exocrine function evaluated by the ^13^C-trioctanoin breath test as a significant physiological predictor of pancreatic fistula after pancreaticoduodenectomy

**DOI:** 10.1186/s12893-022-01500-7

**Published:** 2022-02-11

**Authors:** Hiroyuki Kato, Yukio Asano, Masahiro Ito, Norihiko Kawabe, Satoshi Arakawa, Masahiro Shimura, Daisuke Koike, Chihiro Hayashi, Kenshiro Kamio, Toki Kawai, Takayuki Ochi, Hironobu Yasuoka, Takahiko Higashiguchi, Daisuke Tochii, Yuka Kondo, Hidetoshi Nagata, Toshiaki Utsumi, Akihiko Horiguchi

**Affiliations:** Department of Gastroenterological Surgery, Fujita Health University School of Medicine, Bantane Hospital, 3-6-10 Otobashi Nakagawa Ward, Nagoya, Aichi Prefecture 454-8509 Japan

**Keywords:** ^13^C-trioctanoin breath test, Pancreatic fistula, Postoperative fat absorption

## Abstract

**Background:**

The association between pancreatic fistula (PF) after pancreaticoduodenectomy (PD) and preoperative exocrine function is yet to be elucidated. This study aimed to evaluate the association between the preoperative results of the ^13^C-trioctanoin breath test and the occurrence of PF, showing the clinical relevance of the breath test in predicting PF.

**Method:**

A total of 80 patients who underwent ^13^C-trioctanoin breath tests prior to PD from 2006 to 2018 were included in this study. Univariate and multivariate analyses were conducted to reveal the preoperative predictors of PF, showing the association between ^13^C-trioctanoin absorption and PF incidence.

**Results:**

Among 80 patients (age, 68.0 ± 11.9 years, 46 males and 34 females; 30 pancreatic ductal adenocarcinoma [PDAC]/50 non-PDAC patients), the incidence of PF was 12.5% (10/80). Logistic regression analysis results revealed that the frequency of PF increased significantly as the ^13^C-trioctanoin breath test value (Aa% dose/h) increased (odd’s ratio: 1.082, 95% confidence interval: 1.007–1.162, p = 0.032). Moreover, the optimal cutoff value of the preoperative fat absorption level to predict PF was 38.0 (sensitivity, 90%; specificity, 74%; area under the curve, 0.78; p = 0.005). Indeed, the incidence of PF was extremely higher in patients whose breath test value was greater than 38.0 (33%, 9/27) compared with that in patients with values less than 38.0 (1.8%, 1/53).

**Conclusions:**

Favorable preoperative fat absorption evaluated using the ^13^C-trioctanoin breath test is a feasible and objective predictor of PF after PD.

## Background

Postoperative mortality after pancreaticoduodenectomy (PD) has been decreasing, especially in high-volume centers, due to advancements in surgical skills and perioperative administration. The 30-day and in-hospital mortality rates were reported to be 1.2% and 2.8%, respectively, according to Japan’s National Clinical Database [1]. However, postoperative pancreatic fistula (PF) is still a significant threat to both patients and pancreatic surgeons because it sometimes causes fatal postoperative intra-abdominal bleeding [2, 3] and abscess [4, 5], and its incidence is still reported to be high (11–29.4%) in patients with soft pancreas [6–8]. Therefore, there is a need to elucidate the global preoperative risk factors for PF, and several studies have shown preoperative risk factors such as obesity, fatty pancreas, narrow pancreatic duct, male sex, and surgical techniques [9–11]. However, most predictors may indirectly affect the incidence of PF. In contrast, we conjectured that preoperative favorable exocrine function, which could be associated with a normal pancreas, directly affects the incidence of PF, due to disruption of the anastomotic site caused by excessive secretion of pancreatic juice after PD. However, this assumption remains unclear because it is still clinically challenging to address preoperative exocrine function, which is mainly represented by fat absorption.

Regarding evaluation of pancreatic exocrine functions, several studies have already reported the relevance of various pancreatic function tests [12–17], including *N*-benzoyl-l-trypsyl-p-aminobenzoic acid, fecal chymotrypsin, and fecal elastase-1 (FE-1) testing; fecal fat excretion test; and ^13^C-trioctanoin breath test. Among these, the ^13^C-trioctanoin breath test does not require urine or stool collection and is not affected by the patient’s hepatorenal function; thus, we consider it possibly more acceptable in evaluating perioperative pancreatic exocrine function. This study aimed to evaluate the association between the results of the preoperative ^13^C-trioctanoin breath test and the occurrence of PF, showing the clinical relevance of the breath test in predicting PF.

## Methods

This study included 80 PD patients who provided preoperative consent for the ^13^C-trioctanoin breath test from 2006 to 2018 and precisely evaluated the perioperative course and factors associated with PF. The ^13^C-trioctanoin breath test, which directly and objectively reflects the ability of fat absorption, was performed one to three times before the operation.

Patients who could consume regular meals before the operation were considered eligible for this study. Patients who could not consume regular meals for any reason or those with symptoms due to gastrointestinal obstruction were excluded because the breath test could not be performed properly. Pancreatic enzymes and other digestive enzymes were discontinued from the day before the test for all the patients. All patients fasted overnight prior to the breath test. Breath samples were collected in 100-mL bags using a one-way check valve. Samples were obtained 15 min prior to the test and 0, 5, 10, 15, 20, 30, 40, 50, 60, 75, 90, 105, 120, 135, 150, 165, 180, 210, and 240 min after oral administration of ^13^C-trioctanoin included in the diet (Lacol 200 kcal/200 mL + fat component, 20 g). Fat absorption was evaluated by Aa (Aa = area under the curve [AUC] ∞ Kel × Vd) [Kel, 0.35; Vd, distribution volume] using POCone®, which is the specific analyzer of ^13^CO_2_ concentration in exhaled air. In the present study, preoperative fat absorption levels were retrospectively compared between the groups with and without PF. The Medical Ethics Committee of the Fujita Health University School of Medicine approved the study protocol (HM17165). In terms of the surgical procedure of PD, the inferior pancreaticoduodenal artery (IPDA) was initially employed to reduce intraoperative blood loss [18]. A drain was removed until postoperative days (PODs) 5 to 7 as long as drain discharge was clear and drain amylase level was not as high as the upper limit of the serum amylase level (132 U/mL).

In all patients, the amylase level of the abdominal drainage fluid was measured until day 7 after PD. PF was defined and graded according to the International Study Group on Pancreatic Fistula classification [19]. In the present study, the subjects were divided into patients with clinically relevant grade B or C PF and those with non-PF or biochemical leak. To identify preoperative and intraoperative risk factors for PF, various factors were compared between the two groups.

**Fig. 1:**
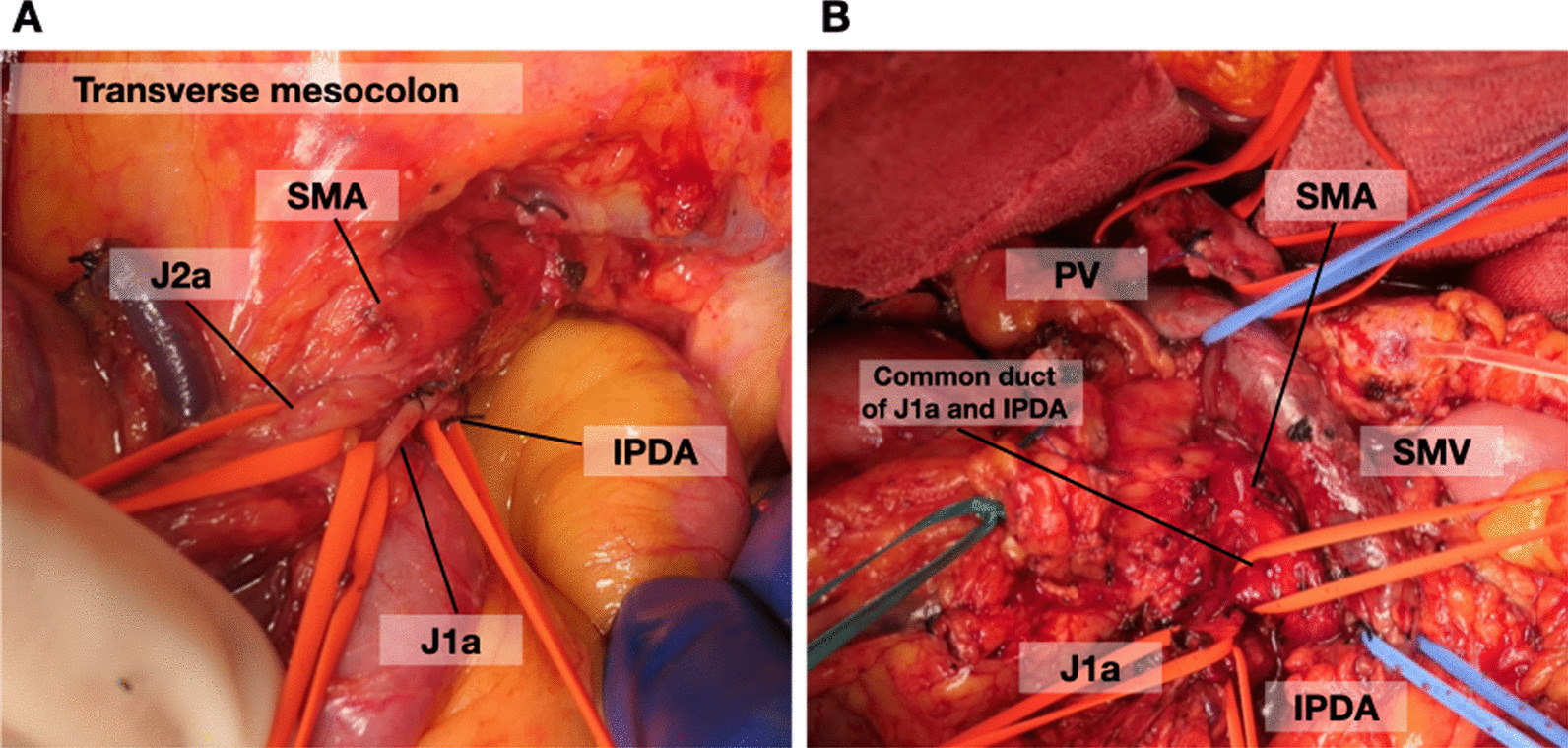
Pancreaticoduodenectomy procedure. **A** IPDA initial approach. Before pancreatic resection, the IPDA is encircled and ligated, reducing the amount of blood loss. **B** The dissection of the SMA plexus during PD. The common duct of the IPDA and J1a is encircled and then transected. *SMA* superior mesenteric artery; *IPDA* inferior pancreaticoduodenal artery; *J1a* first jejunal artery; *J2a* second jejunal artery; *PV* portal vein; *SMV* superior mesenteric artery

In terms of the surgical procedure of PD, the IPDA approach was employed from 2007 [18]. Briefly, IPDA was encircled and ligated before pancreatic resection to reduce intraoperative blood loss (Fig. 1). For pancreatojejunostomy, the first-layer anastomosis was performed through duct-to-mucosa anastomosis with 6–8 interrupted sutures using 5-0 PDS II (Ethicon, Inc., Somerville, NJ, USA). The second-layer anastomosis was performed using the modified Kakita procedure with six sutures using 3-0 Prolene [20]. A 5-F external pancreatic stent tube was inserted into the remnant main pancreatic duct in all 80 patients.

All statistical analyses were performed using the statistical software package SPSS for Macintosh (version 24.0, IBM, Armonk, NY, USA). Logistic regression analysis was performed to determine the association between the results of the preoperative breath test and the incidence of PF. Pre- and intraoperative risk factors associated with PF were analyzed using univariate and multivariate analyses. The results of the continuous variables are expressed as medians and ranges, and statistical significance was evaluated using the Mann–Whitney U test. Discrete variables were evaluated through χ^2^ analysis or Fisher’s exact test, as appropriate. Only variables with p-value of < 0.05, as determined by univariate analysis, were included in multivariate analysis by logistic regression analysis. p-values of < 0.05 were considered statistically significant. Receiver operating characteristic (ROC) curve analysis was employed to estimate the optimum cutoff points for the ^13^C-trioctanoin breath test to predict PF.

## Results

The preoperative backgrounds of the 80 patients are shown in Table 1. In these patients, the median age (range) was 69.5 (26–88) years old, and the number of males and females was 46 and 34, respectively. The primary disease was pancreatic ductal adenocarcinoma (PDAC) in 30 patients and non-PDAC in 50 patients.

**Table 1:** Background of 80 patients who underwent pancreaticoduodenectomy

	PD (n = 80)
Pre-operative variables	
Age (years)	69.5 (26–88)
Gender (male/female)	46/34
Body weight (kg)	51.5 (33.0–96.0)
Diagnosis PDAC/non PDAC	30/50
Hemoglobin (g/dl)	12.7 (8.7–15.9)
White blood cell counts (/mm^2^)	5200 (620–11,300)
Neutrophil (/mm^2^)	3200 (1365–8512)
Lymphocyte (/mm^2^)	1470 (400–2940)
Total protein (mg/dl)	6.9 (5.7–8.3)
Albumin (mg/dl)	4.0 (2.5–5.0)
Serum amylase (U/l)	90 (12–604)
Total cholesterol	182 (120–282)
Breath test (%dose/h)	34.4 (16.4–69.7)
Intra-operative variables	
Operation time (min)	469.0 (296–842)
Blood loss (g)	325 (23–4900)
Diameter of main pancreatic duct	3.0 (1.3–12.3)
Pancreatic texture (soft/hard)	46/34
Post-operative variables	
Pancreatic fistula (yes/no)	10/70

*PDAC* pancreatic ductal adenocarcinoma

In terms of preoperative laboratory data, the details of blood cell counts and several nutritional markers are described in Table 1. The median operation time (min) and intraoperative blood loss (mL) were 469 (296–842) min and 325 (23–4,900) g, respectively. Regarding the incidence of PF, clinically relevant PF (more than grade B according to the International Study Group Pancreatic Fistula criteria) was found in 10 out of 80 (12.5%) patients. With regard to the results of breath test, the values of Aa before PD were 34.4 (16.4–69.7) dose/h.

**Fig. 2:**
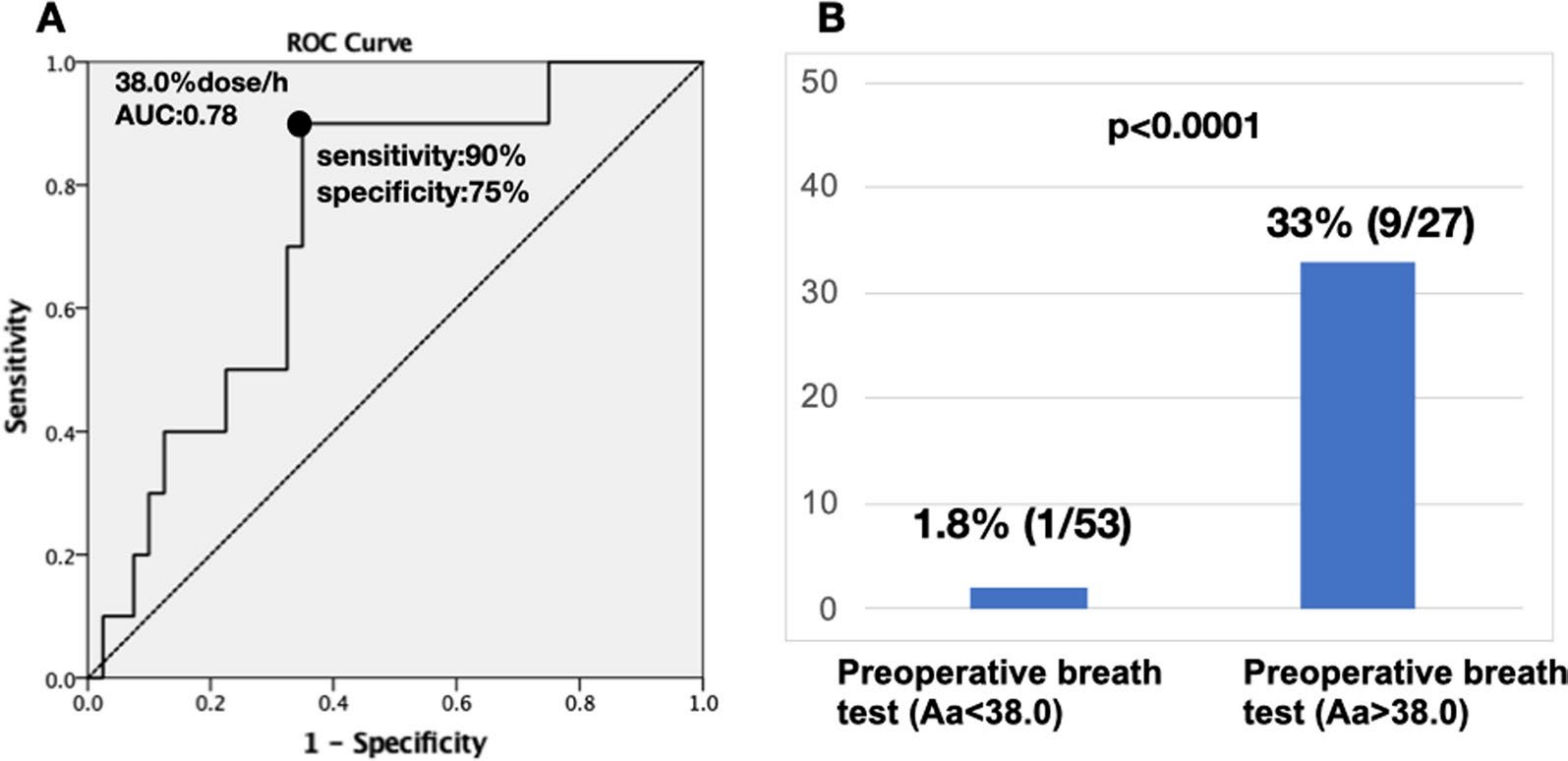
**A** Receiver operating characteristic (ROC) curve. The cutoff point of the 13C breath test (Aa) is 38.0% dose/h (AUC: 078). **B** The incidence of POPF according to the cutoff value (Aa)

First, we assessed how the results of the preoperative breath test affected the frequency of postoperative PF and found that the frequency of PF increased significantly as the ^13^C-trioctanoin breath test value (Aa % dose/h) increased (odd’s ratio: 1.082, 95% confidence interval: 1.007–1.162, p = 0.032). The optimal cutoff value of the preoperative fat absorption level to predict PF was 38.0 (sensitivity, 90%; specificity, 75%; AUC, 0.78) according to the ROC curve (Fig. 2A). Indeed, the incidence of PF was extremely higher in patients whose breath test value was greater than 38.0 (33%, 9/27) compared with that in patients with values less than 38.0 (1.8%, 1/53) (Fig. 2B).

Second, to determine the superiority of the breath test over other preoperative predictors of PF, univariate and multivariate analyses were conducted. As shown in Table 2, univariate analysis comparing preoperative risk factors between the PF and non-PF groups identified PDAC (p = 0.009), soft pancreatic texture (p = 0.038), and high levels of the ^13^C-trioctanoin breath test (p = 0.005) as significant risk factors for PF. Indeed, when comparing the levels of ^13^C-trioctanoin absorption between the PF and non-PF groups, the preoperative fat absorption level was significantly higher in the PF group than that in the non-PF group (40.2 vs. 34.4, p = 0.005).

**Table 2:** Univariate analysis for identifying risk factor of PF

	Non-PF (n = 70)	PF (n = 10)	p-value
Pre-operative variables			
Age (years)	69.0 (43–88)	71.0 (26–79)	0.961
Gender (male/female)	38/32	8/2	0.114
Body weight (kg)	50.6 (33.0–83.5)	63.9 (44.3–96.0)	0.075
Diagnosis PDAC/non PDAC	30/40	0/10	*0.009
Hemoglobin (g/dl)	12.8 (8.9–15.6)	12.1 (9.1–15.9)	0.708
White blood cell counts (/mm^2^)	5100 (3300–11,300)	4950 (2100–8000)	0.782
Neutrophil (/mm^2^)	3245 (2046–8512)	2718 (1365–4880)	0.106
Lymphocyte (/mm^2^)	1428 (630–2438)	1760 (400–2940)	0.135
Total protein (mg/dl)	6.9 (4.7–8.3)	6.6 (6.7–7.8)	0.923
Albumin (mg/dl)	4.0 (2.5–4.7)	4.0 (2.7–5.0)	0.857
Serum amylase (U/l)	90 (12–604)	94.5 (45–218)	0.903
Total cholesterol	179 (120 -282)	195 (120–282)	0.295
Breath test (%dose/h)	33.4 (16.3–69.6)	40.2 (29.1–51.4)	*0.005
Intra-operative variables			
Operation time (min)	466.5 (296–842)	489.0 (338–607)	0.813
Blood loss (g)	325.5 (23–4900)	321.0 (205–1545)	0.745
Diameter of main pancreatic duct	3.2 (1.3–12.3)	2.3 (1.8–5.7)	0.125
Pancreatic texture (soft/hard)	37/33	9/1	*0.038

*PF* pancreatic fistula, *PDAC* pancreatic ductal adenocarcinoma

By multivariate analysis (Table 3), preoperative ^13^C-trioctanoin breath test values > 38.0% dose/h were selected as the most independent risk factor for PF (p = 0.001; odds ratio, 16.7).

**Table 3:** Results of multivariate analysis for identifying risk factor of PF

Variables	Odd’s ratio	95% CI	p-value
PDAC	Not applicant*	0.00–0.00	0.998
Soft pancreatic texture	1.46	0.121–17.61	0.766
Preoperative breath test (> 38)	17.1	1.948–150.26	0.010

There is no patients with PF in PDAC group

*PDAC* pancreatic ductal adenocarcinoma

**Fig. 3:**
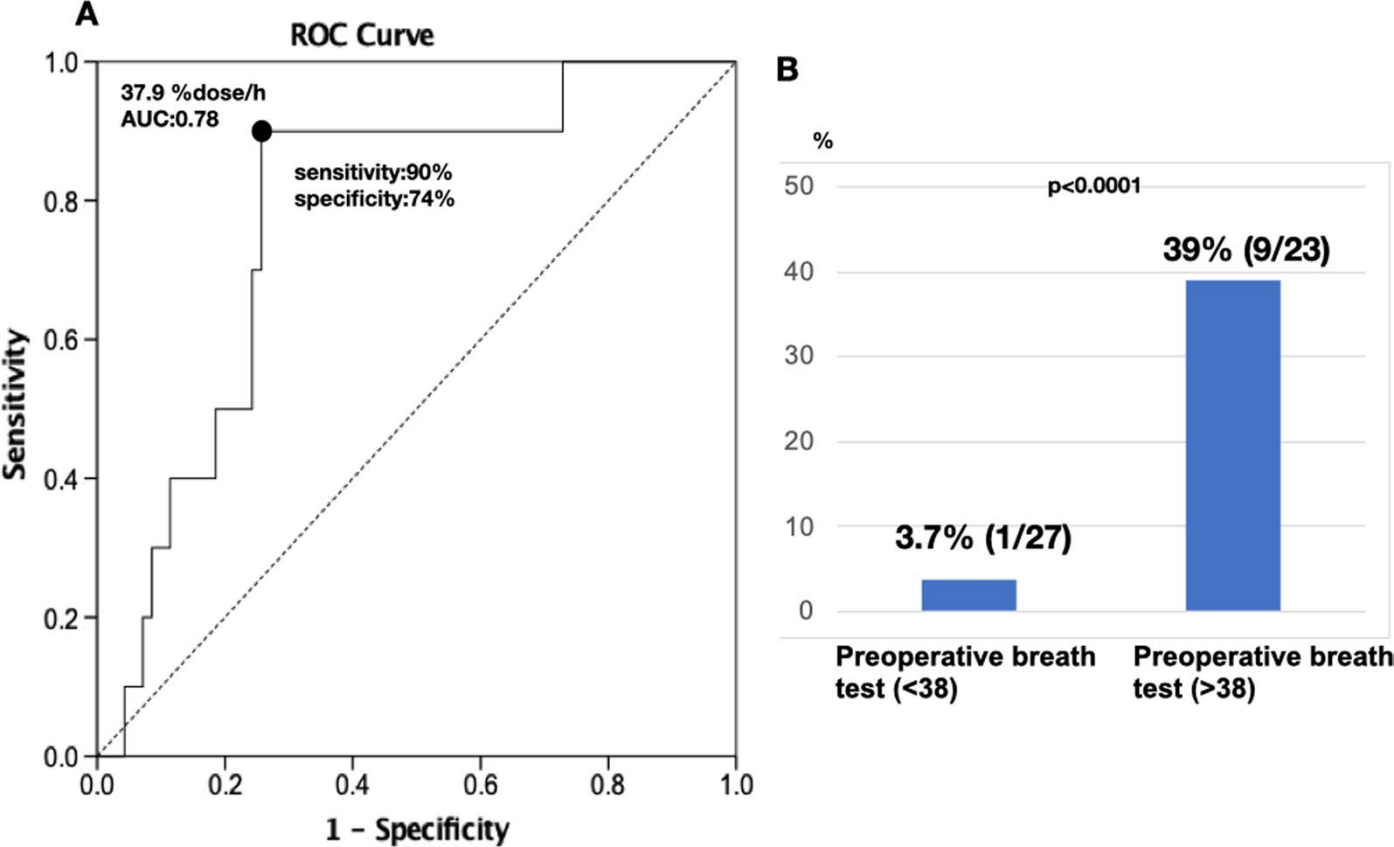
**A** Receiver operating characteristic (ROC) curve predicting PF in 50 non-PDAC patients. The cutoff point of the 13C breath test (Aa) is 37.9% dose/h (AUC: 078). **B** The incidence of POPF according to the cutoff value (Aa %dose/h). The dissection of the SMA plexus during PD. The common duct of the IPDA and J1a is encircled and then transected. *SMA* superior mesenteric artery; *IPDA* inferior pancreaticoduodenal artery; *J1a* first jejunal artery; *J2a* second jejunal artery; *PV* portal vein; *SMV* superior mesenteric artery

**Fig. 4:**
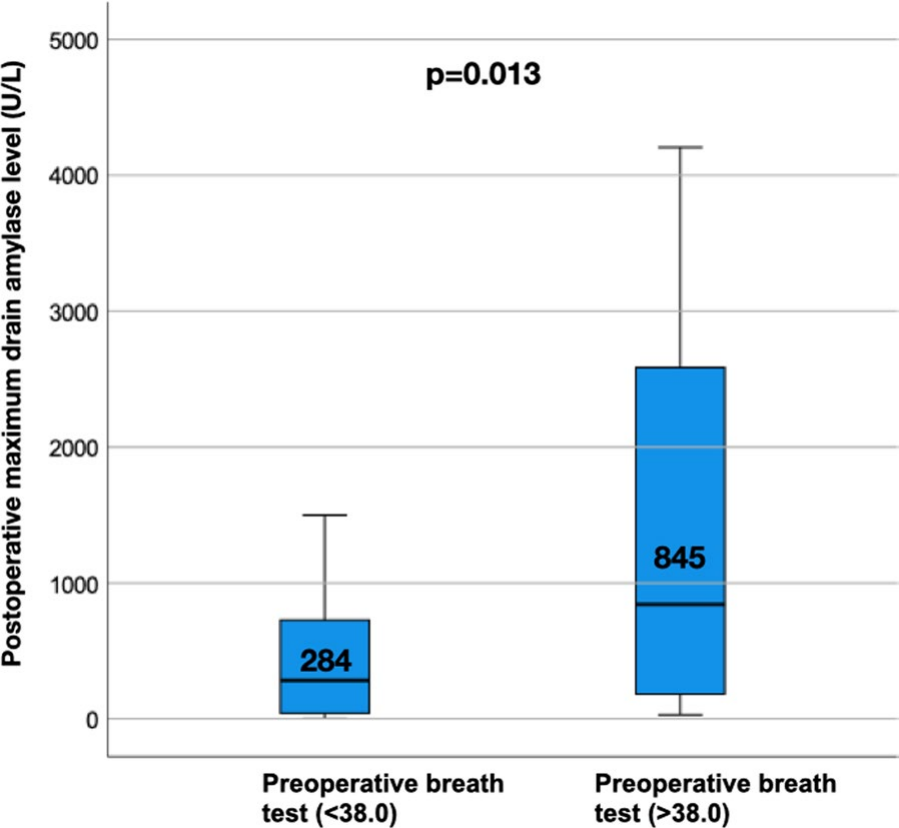
Comparison of postoperative maximum drain amylase level (U/L) according to the result of the preoperative breath test

Moreover, we focused on the association between the incidence of PF and the ^13^C-trioctanoin breath test value of > 38.0% dose/h in non-PDAC patients only, considering that the prediction of PF in non-PDAC cases, which are mostly soft pancreas, is an urgent issue to be solved. As shown in Fig. 3A, the ROC curve revealed that the cutoff value was 37.9% dose/h (AUC: 0.78). The incidence of PF was markedly high (39.0%, 9/23) in patients with favorable preoperative fat absorption, whereas it was 3.7% (1/27) in patients with unfavorable absorption (Fig. 3b). When the postoperative maximum drain amylase level (U/L) was compared based on the results of the preoperative breath test, the drain amylase levels were significantly higher in patients with Aa > 38.0% dose/h than in patients with Aa < 38.0 dose/h (Fig. 4).

## Discussion

In the present study, we found that ^13^C-trioctanoin absorption (> 38.0% dose/h) was a strong preoperative physiological predictor of PF after PD in not only the overall cohort, but also in non-PDAC patients whose pancreatic parenchyma could be soft.

^13^C-trioctanoin breath tests have been employed in clinical settings to evaluate pancreatic exocrine deficiency by detecting fat malabsorption through the gut after pancreatectomies [21, 22]. Till now, however, there have been few studies evaluating whether preoperative exocrine function tests affect the incidence of PF after PD [12, 23].

To predict the development of PF preoperatively, many researchers have sought to identify the relevant risk factors such as high body mass index, fatty pancreas [10, 24, 25], male sex [26, 27], and untreated jaundice [28] and developed a method for its prediction using various imaging modalities such as computed tomography (CT) configurations (narrow main pancreatic duct [10], thick pancreatic parenchyma [10], pancreatic border [6], CT attenuation value [29]), Magnetic resonance imaging (MRI) findings [30], and pancreatic ultrasound elastography [31, 32]. Although these predictors might be clinically useful, most of these factors are strongly associated with the soft parenchymal condition, which causes technical difficulty during anastomosis. Therefore, whether these risk factors are reproducible is also dependent on the type of pancreato-enteral anastomosis and the maturity of those procedures. Conversely, data obtained from the ^13^C-trioctanoin breath test are more objective and quantitative, and especially in patients with Aa > 38.0% dose/h, the PF incidence is extremely high regardless of the parenchymal condition. A previous study reported that the recovery of ^13^C-trioctanoin absorption after PD was positively associated with the output of pancreatic enzymes such as lipase, amylase, and chymotrypsin [15]. Thus, we speculated that the active production of pancreatic juice might be one of the major causes of PF after PD and considered that the outcome of the present study represented this aspect. In fact, our speculation is supported by the result showing that the postoperative maximum drain amylase level (U/L) was significantly higher in patients with Aa > 38.0% dose/h than in those with Aa < 38.0% dose/h (Fig. 4).

Previous reports evaluating the association between PF and the results of pancreatic exocrine function tests revealed that higher preoperative levels of FE-1 were positively associated with the development of postoperative PF [12, 29]. However, there has been no study revealing the association between the results of ^13^C-trioctanoin breath test and the occurrence of PF. Therefore, to the best of our knowledge, Aa of > 38.0% dose/h can be considered the first physiological quantitative predictor of PF.

The clinical application of this study is challenging because prevention of PF is quite difficult even if high-risk patients are identified preoperatively. The administration of octreotide or somatostatin analogs is a well-accepted pharmacological treatment of PF targeting the secretion of pancreatic juice [33, 34]. Somatostatin analogs reduce the volume of fistula output, thereby potentially alleviating PF [35]. Octreotide has also been considered to reduce the volume and potency of both pancreatic exocrine secretions and hormone production [36]. Since our study demonstrated that favorable preoperative exocrine function, which produces high pancreatic juice output, is regarded as a risk factor for PF, administration of these drugs might become a key treatment for PF in these high-risk patients. However, prospective or randomized control studies are required to confirm this hypothesis.

The present study has several limitations. First, it included only a small number of patients who provided preoperative consent, and subject are not consecutive. Second, this was a retrospective analysis, and the precise mechanism by which favorable pancreatic exocrine function causes PF postoperatively could not be identified. Therefore, this study is regarded as exploratory research. Nonetheless, our study could draw significant attention to the association between PF and preoperative pancreatic exocrine function.

## Conclusions

In conclusion, favorable pancreatic exocrine function evaluated by the ^13^C-trioctanoin breath test preoperatively is a feasible and objective predictor of PF after PD, paying attention to the development of PF in high-risk patients.

## Data Availability

The datasets analyzed during the current study are available from the corresponding author upon reasonable request.

## Abbreviations

AUC: Area under the curve
FE-1: Fecal elastase-1
IPDA: The inferior pancreaticoduodenal artery
PD: Pancreaticoduodenectomy
PDAC: Pancreatic ductal adenocarcinoma
PF: Pancreatic fistula
POD: Postoperative day
ROC: Receiver operating characteristic
